# Risk Assessment and Source Apportionment of Soil Heavy Metals under Different Land Use in a Typical Estuary Alluvial Island

**DOI:** 10.3390/ijerph17134841

**Published:** 2020-07-05

**Authors:** Ting Sun, Jingling Huang, Yuying Wu, Yuan Yuan, Yujing Xie, Zhengqiu Fan, Zhijian Zheng

**Affiliations:** Department of Environment Science and Engineering, Fudan University, Shanghai 200433, China; sunt17@fudan.edu.cn (T.S.); 18210740006@fudan.edu.cn (J.H.); 18210740063@fudan.edu.cn (Y.W.); 17210740019@fudan.edu.cn (Y.Y.); xieyj@fudan.edu.cn (Y.X.)

**Keywords:** soil heavy metal, risk assessment, source apportionment, positive matrix factorization, land use

## Abstract

Understanding the environmental risks of soil heavy metals (HMs) and identifying their main sources are the essential prerequisites for the prevention and management of soil pollution. Based on a detailed survey of soil HMs (Cu, Cr, Ni, Zn, Pb, Cd, As and Hg) from different land use types (including agricultural land, construction land, wetland, and forest land) in an estuary alluvial island, the environmental risk and source apportionment of soil HMs were investigated. Altogether, 117 soil samples were taken in the study area to appraise the soil HMs environmental risk by using the geo-accumulation index (Igeo), potential ecological risk index (RI), and human health risk assessment (HRA) and to identify its main sources by using positive matrix factorization (PMF) model. The average concentrations of soil HMs (except As) surpassed their reference background values in China. There were no significant differenced in the mean concentrations of HMs in different land use types, except that the Hg concentration in the construction land was significantly higher than that in others. The results of Igeo showed that Cd pollution was unpolluted to moderately polluted, and that the others were unpolluted. The potential ecological risk level for Cd and Hg was “moderated potential risk”, while for Cu, Cr, Ni, Zn, Pb and As was “low potential risk”. Higher contamination was distributed at the west-central area. The results of the HRA indicated that the non-carcinogenic risk and the carcinogenic risk that human beings suffered from HMs in different land uses were insignificant. To more accurately identify the sources of soil HMs, the PMF model coupled with the GIS-spatial analysis was applied. The results showed that agricultural activities, natural source, industrial discharge and river transportation, and atmosphere deposition were the main determining factors for the accumulation of soil HMs in the study area, with the contribution rate of 24.25%, 23.79%, 23.84%, and 28.12%, respectively. The study provides an underlying insight needed to control of the soil HM pollutions for an estuary alluvial island.

## 1. Introduction

In the process of urbanization and industrialization, the emission of various pollutants has caused serious environmental pollution, especially the heavy metal (HM) pollution [1]. Due to its toxic effects, persistence and bioaccumulation in environment, the HM pollution has attracted widespread attention, among which the HM distribution in soil is particularly relevant to humans [2]. According to the United States Environment Protection Agency (US EPA), Ministry of Environmental Protection of the People’s Republic of China (MEPER), and the World Health Organization (WHO), the accumulation of the soil HMs not only results in soil degradation and reduces the quantity and quality of crops, but also can do harm to human health (e.g., headache, insomnia, insanity, joint pain, and cancer) through different exposure pathways (such as direct ingestion via food chain transmission, inhalation via mouth and nose, and direct dermal contact) [3,4,5]. Therefore, it is meaningful to estimate the contamination and human health risk of soil HMs for improving the environment and protecting human health.

As a comprehensive reflection on various human activities, land use is an important factor affecting metal distribution in the soil. According to the different ways of land use, land can generally be divided into construction land, farmland, wetland, residential land, forest land, industrial land, etc. [6]. Previous studies have indicated that long-term fertilizer and pesticide application can result in a higher content of Cu, Cd, and Zn in agricultural soils [7]. The construction land, which is strongly influenced by anthropogenic inputs (such as chemical industry, vehicle exhaust discharge, and coal combustion) can result in high levels of Cd, Cu, and Pb in the soil [8]. On the other hand, the forest land is generally less influenced by human inputs and its HM concentration is relatively low [9]. Therefore, the sources of HM pollution would be different in different land use types. Many studies have investigated the contamination level, environmental risk or pollution sources of soil HMs in single land use type, such as agricultural land [7], forest land [10], industrial land [11], residential land [8], etc. Nevertheless, few studies are procurable on the relation between the environmental risk caused by soil HMs and land use types.

In addition to natural sources, the different concentrations and distribution of HMs in soils may also be caused by the different human activities, such as transportation emissions, industrial emission, urban sewage, atmospheric sedimentation, and agricultural activities [12]. Identifying the potential sources of soil HMs is important for controlling the priority pollutants. Recently, various mathematical models and statistical analysis methods, such as enrichment factors (EFs), cluster analysis (CA), principal component analysis (PCA) and so on, have been applied to identify the sources of soil HMs. Ye et al. (2011) used EFs to distinguish the natural and human origins of soil HMs in the Three Gorges Reservoir [13]. Tume et al. (2019) used CA and PCA to distinguish the potential sources of soil metals in Hualpen, Chile [2]. Lv et al. (2015) used geostatistical analysis and PCA to discriminate the sources of HMs in soil of Ju country (Eastern China) [14]. Luo et al. (2015) employed the isotope labeling to distinguish specific Pb contributors [15]. However, the source apportionment of many studies was only a qualitative analysis for the pollution sources based on professional experience and models, which cannot quantify the contribution rate of various sources. In recent years, several source identification methods, like the absolute principal component scores–multiple linear regression (APCS–MLR), chemical mass balance (CMB), and positive matrix factorization (PMF), have made contributions to the field of the quantification of pollution sources [16,17]. Among them, the PMF model has a better ability of source analysis, because it can carry out non-negative constraints on factor load and factor score in the analysis process, and quantify the contribution rate of each source to each HM. In particular, to more accurately identify the sources of HM pollution, the GIS spatial analysis combined with the PMF model was applied to analyze and quantify the potential sources of soil HMs in this study.

Many previous studies on the HM pollution have focused on cities, the industrial area, and mining areas, etc. [11,18,19]. Little research has concentrated on islands. In this study, Chongming Island, the largest estuary alluvial island in the world, was selected as a case study area. Based on the information from the Shanghai statistical yearbook, compared with the highly urbanized Shanghai metropolis in China, Chongming Island has the characteristics of low population density, low urbanization, and low development intensity, speculating that its pollution sources and environmental risk of soil HMs may be completely different from that of Shanghai (Figure S1) [20]. Thus, the main purposes of this study were to (1) investigate the characteristics of soil HMs in the study area; (2) to compare the differences in the ecological risks and the human health risks of soil HMs under different land use; (3) to identify and quantify the potential pollution sources of soil HMs by using a PMF model coupled with GIS spatial analysis.

## 2. Materials and Methods

### 2.1. Study Area

**Figure 1:**
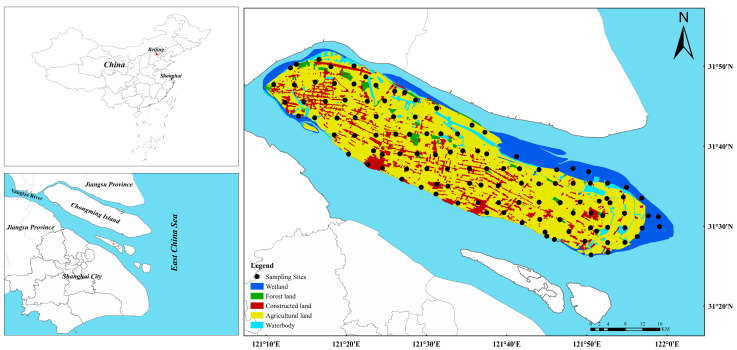
The map of the study area and the distribution of the sampling sites.

Chongming Island (31.45°−31.85°N, 121.16°−121.90°E) is the largest estuary alluvial island in the world with an overall area of approximately 1515 km^2^ and a population of 690 thousand (Figure 1). It is located at the intersection of the Yangtze River and the East China Sea and developed from the alluvium of the Yangtze River in modern times. It belongs to the subtropical monsoon climate with the mean temperature of 15.2 °C and the mean yearly rainfall of over 1000 mm. According to the results of the graphic interpretation of remote sensing images which originated from the National Earth System Science Data Sharing Infrastructure of China and Resource (http://www.geodata.cn/) and the Environment Data Cloud Platform (http://www.resdc.cn/data.aspx?DATAID=264), the main types of land use in the study area are divided into agricultural land, construction land, wetland and forest land, accounting for 65.27%, 8.98%, 14.32%, and 2.26%, respectively (Figure 1). It is worth noting that this area is the largest agricultural planting base in Shanghai, and its agricultural products are mainly supplied to the whole Shanghai area. At present, Chongming Island is a key ecological protection area in Shanghai, and the population density, and urbanization level in this area are the lowest among all the districts in Shanghai (Figure S1). The main economic activities of Chongming Island are ecological agriculture, tourism and marine equipment.

### 2.2. Sample Collection and Analysis

Altogether, 117 topsoil samples (0–20 cm) were taken in Chongming Island from April to June 2019, including 51 agricultural land sampling sites, 33 forest land sampling sites, 17 wetland sampling sites, and 16 construction land sampling sites (Figure 1). The soil sampling was primarily conducted using a 4 × 4 km grid, and the density was adjusted according to the topography and land use type. After homogeneous mixing, each soil sample was composed of five sub-samples of the sampling site, and the geographic coordinates of the sampling point were located and recorded simultaneously by Global Positioning System (GPS). The samples were placed in clear polypropylene (PP) plastic bags and sent to the laboratory for analysis.

After air-drying at room temperature, all the soil samples were sieved with a 100 mesh nylon sieve [21]. The parameters of pH, cation exchange capacity (CEC), total nitrogen (TN), total phosphorus (TP) and organic carbon (OC) were detected in the laboratory with the method of Liu et al. (2017). For the metal analysis, 0.25 g of sample was placed in a Teflon bomb with an acid mixture (5:4:1 HNO_3_+HClO_3_+HF) and then heated to 120 °C for 12 h on a heating plate. The acid digestion was repeated until only a negligible amount of white residue remained. Afterwards, the solution was evaporated to dryness and extracted. Following the complete digestion, the solution was sieved through a Whatman paper, and the Cu, Cr, Ni, Zn, Pb and Cd concentrations in the soil were analyzed by inductively coupled plasma mass spectrometry (ICP–MS, 7900, Thermo Fisher, Waltham, MA, USA). Additionally, the As and Hg concentrations were measured using an atomic fluorescence spectrometer (AFS, AFS−8220, Beijing Titan Instruments, Beijing, China). According to the method of inductively coupled plasma–mass spectrometry (USEPA 6020B−2014), the detection limits for Cu, Cr, Ni, Zn, Pb and Cd were 0.1, 0.1, 0.1, 0.5, 0.1, and 0.01 mg/kg, respectively. According to the method of the soil quality analysis of total mercury, arsenic and lead contents–atomic fluorescence spectrometry (GB/T 22105–2018), the detection limits for As and Hg were 0.01 and 0.002 mg/kg, respectively.

Adequate quality assurance (QA) and quality control (QC) measures were strictly implemented throughout the study. Reagent blanks and three analytical duplicates were implemented to monitor accuracy and precision. Guaranteed reagent and the de-ionized water was applied throughout the study. Certified reference samples were purchased from the National Research Center for Certified Reference Materials (CRMS) (Beijing, China). The recovery rates of the standard addition were between 93% and 110%, and the standard deviations of the parallel tests ranged from 1.27% to 10.52%.

### 2.3. Geo-Accumulation Index (Igeo)

To evaluate the degree of soil HM pollution, a common method was used to calculate the *I_geo_* [22]. This index was calculated as follows:

(1) $$I_{geo}=\log_{2}\left[\frac{C_{n}}{1.5B_{n}}\right]$$

where $C_{n}$ is the measured concentration of HM *n* in soil, and $B_{n}$ is the background value of the HM *n*. A factor of 1.5 was introduced to reduce the possible fluctuations in the background values, which might be caused by diagenesis [23,24]. The background value for Cu, Cr, Ni, Zn, Pb, Cd, As and Hg was 22.6, 61, 26.9, 74.2, 26, 0.097, 11.2 and 0.065 mg/kg, respectively [25].The degree of pollution was classified into seven enrichment classes (Table S1).

### 2.4. Potential Ecological Risk Index (RI)

Based on the content, the toxicity and the background values of the HMs, the *RI* put forward by Hakanson (1980) [26] was applied to appraise the ecological risk posed by the soil HMs. The RI was determined by Equations (2)–(4):

(2) $$C_{f}^{i}=\frac{C^{i}}{C_{b}^{i}}$$

(3) $$E_{r}^{i}=T_{r}^{i}\times C_{f}^{i}$$

(4) $$\mathrm{RI}=\overset{n}{\underset{i}{\sum }}E_{r}^{i}$$

where $C_{f}^{i}$ means the pollution factor of the studied HM i, $C^{i}$ is the measured concentration of HM i in the soil, and $C_{b}^{i}$ is the background value of the HM i [25]. *E_r_^i^* is the potential ecological risk index of the single element. $T_{r}^{i}$ is the biological toxic factor for HM i. The biological toxic factor for Cu, Cr, Ni, Zn, Pb, Cd, As and Hg is 5, 2, 5, 1, 5, 30, 10 and 40, respectively [26]. The grades of the $E_{r}^{i}$ and RI are showed in Table S2.

### 2.5. Human Health Risk Assessment (HRA)

Recently, the HRA has been extensively applied to evaluate the hazards of soil HMs to human beings, including non-carcinogenic risk (NCR) and carcinogenic risk (CR) according to the USEPA Health Risk Handbook and the Technical Specification for Soil Monitoring [3,4]. Soil HMs might do harm to the human health by the three exposure routes: (1) direct ingestion; (2) inhalation through mouth and noise; and (3) direct dermal contact (Huang et al. 2018). The HRA commended by the US EPA [27] was used in this study. The average daily doses (*ADDs*) (mg/kg-day) of HMs via ingestion ($ADD_{ing}$), inhalation ($ADD_{inh}$) and dermal contact ($ADD_{derm}$) for both adults and children were evaluated by the following Equations (5)–(7):

(5) $$ADD_{ing}=\frac{c\times IngR\times CF\times EF\times ED}{BW\times AT}$$

(6) $$ADD_{inh}=\frac{c\times InhR\times EF\times ED}{PEF\times BW\times AT}$$

(7) $$ADD_{derm}=\frac{c\times SA\times CF\times AF\times ABS\times EF\times ED}{BW\times AT}$$

The definition and reference value of the parameters used in Equations (5)–(7) are referred to in Table S3.

In this study, the NCR and CR of the HMs were evaluated using a hazard quotient (HQ), total hazard quotient (THQ), carcinogenic risk (CR) and the total carcinogenic risk (TCR). The HQ is the ratio of the *ADD* of a HM to its reference dose (RfD) (mg/kg-day) for the same exposure pathways. The CR is the product of the ADD and its carcinogenic slope factor of pollutants (SF). The THQ and TCR are calculated by Equations (8)–(11):

(8) $$\mathrm{HQ}=\frac{ADD}{RfD}$$

(9) $$\mathrm{THQ}=\sum HQ_{i}$$

(10) CR=ADD×SF

(11) $$\mathrm{TCR}=\sum CR_{i}$$

The reference value of *RfD* and SF used in Equations (8)–(11) are referred to in Table S4.

When THQ < 1, it indicates that the NCR is negligible; otherwise, a health risk exists. If the TCR > 1 × 10^−4^, it means that the cancer risk influences human health; if the TCR < 1 × 10^−6^, it means that the CR is negligible; if 1 × 10^−6^ < TCR < 1 × 10^−4^, it means an acceptable cancer risk to human health [28].

### 2.6. Positive Matrix Factorization (PMF) Model

The PMF model, which was produced by Paatero and Tapper (1993) [29], was adopted to the source the apportionment and uncertainty analysis. In order to avoid negative values in the matrix factorization process, the PMF makes non-negative constraints on factor loading and the factor score in solving the process which makes the source component spectrum and source contribution interpretable and with clear physical significance [30,31]. The PMF model can be represented by the following equation:

(12) $$X_{ij}=\overset{p}{\underset{k=1}{\sum }}g_{ik}f_{kj}+e_{ij}$$

where $X_{ij}$ is the concentration of element j in sample i, $g_{ik}$ is the contribution of source k on sample i; $f_{kj}$ is the contribution of source k on element j; and $e_{ij}$ is the modeling error on the concentration of element j in sample i. The minimum value of the objective function Q can be computed by the following formula:

(13) $$Q=\overset{n}{\underset{i=1}{\sum }}\overset{m}{\underset{j=1}{\sum }}{\left(\frac{x_{ij}-\sum_{k=1}^{p}g_{ik}f_{kj}}{u_{ij}}\right)}^{2}$$

where $u_{ij}$ is the uncertainty of element j in sample i.

The remarkable feature of the PMF is requiring the uncertainty to analyze the quality of every concentration data individually [32]. The uncertainty of the concentration was calculated as follows:

If c < method detection limit (MDL):

(14) $$\mathrm{Unc}=\frac{5}{6}MDL$$

Else:

(15) $$\mathrm{Unc}=\sqrt{{\left(Error\;fraction\times c\right)}^{2}+{\left(\frac{MDL}{2}\right)}^{2}}$$

where c is the concentration of the heavy metal; MDL is the species-specific method detection limit; and Error fraction is a percentage of the measurement uncertainty.

### 2.7. Statistical Analysis and Geochemical Mapping

Descriptive statistics were used to describe the HM concentration and distribution in the soils. Prior to analysis, a normality test (skewness–kurtosis tests) was used to assess whether the original data satisfied the normal distribution by the Stata MP (NCSA, Urbana, IL, USA). Box–Cox transformation was carried out for the part of data that did not satisfy the normal distribution to make all the data satisfy the normal distribution by the Stata MP. Before the PMF analysis, according to the interquartile ranges and stem, the dataset was detected and the outliers were excluded to prevent these data from impacting the PMF results (Guan et al. 2018). The PMF was used to assess the relationship among the HMs and identify the possible sources of soil pollution by using the EPA PMF 5.0 (US EPA, Washington, DC, USA). The statistical analysis was achieved by the SPSS v22.0 (IBM, Chicago, IL, USA) and Microsoft Excel 2016 (Microsoft Corporation, Redmond, WA, USA), and the box chart was constructed using the Origin 9.1 software package (ESRI, Redlands, CA, USA). The inverse distance weight (IDW) method was adopted to simulate and predict the spatial distribution in soil as an interpolator using the ArcGIS v10.2 (ESRI, Redlands, CA, USA).

## 3. Results and Discussion

### 3.1. General Statistics of Soil HMs

As the results of descriptive statistics in Table 1, the Cu, Cr, Ni, Zn, Pb, Cd, As and Hg concentrations of soils in the study area were 10.90–104.00, 55.10–134.20, 21.90–51.30, 51.40–210.00, 16.20–195.00, 0.080–0.394, 4.54–17.24, and 0.009–0.244 mg/kg, respectively. The order of the average HM concentrations (mg/kg) was as follows: Zn (95.56) > Cr (87.96) > Ni (34.68) > Cu (30.22) > Pb (29.95) > As (9.14) > Cd (0.208) > Hg (0.077). In addition, compared with the upper continental crust (UCC) (Wedepohl 1995) and world background [1], most soil HMs in the study area showed a certain degree of accumulation (Table 1). Compared to the background values in China [25], the average HM concentrations in the soils except As exceeded their reference background values. However, based on the to the Soil Environmental Quality Management Standard in China (GB 15618–2018), the average concentrations for soil HMs met its reference value in standard level (Table 1). The average contents of most HMs in the study area are below that in the urban zone in Shanghai [21], indicating the soil HM pollution in the study area is lower than that in the urban zone of Shanghai. This may be highly relevant to the discrepancy in population size, industrial development, and urbanization between the study area and the urban zone of Shanghai.

The coefficients of variation (CV) was often used to reflect the discreteness of the observed values of each index on the unit mean. The larger the CV value, the higher the variability (Table S6). As shown in Table 1, the CV values of all HMs followed the order of Pb (66.8%) > Hg (48.9%) > Cu (37.2%) > Cd (29.5%) > Zn (26.9%) > As (26.7%) > Ni (15.6%) > Cr (14.0%). The high CV value for Pb indicated a high variability and suggested that the sources of HMs could come from external factors.

### 3.2. Statistical Analysis of Soil HMs under Different Land Use

**Figure 2:**
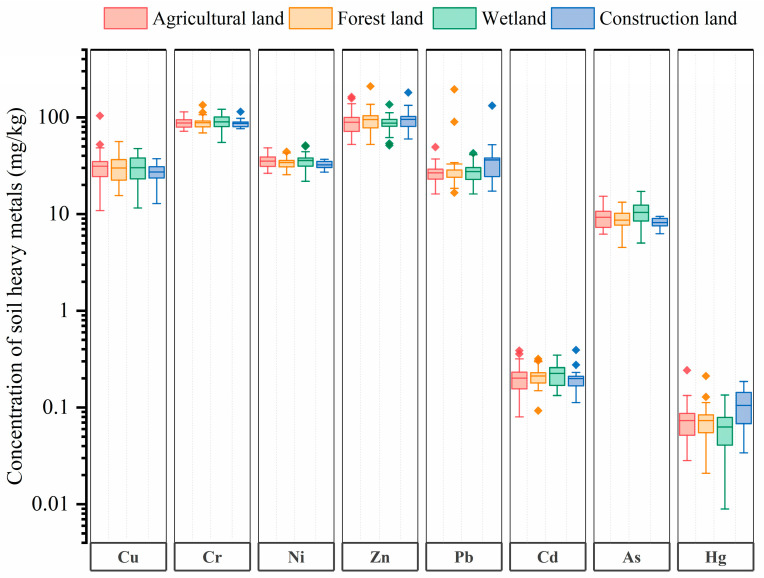
The concentrations of the soil HMs under the different land use types. The scale of the Y axis is logarithmic.

The descriptive statistics analysis (regarding the mean, min, max, SD, and CV) for all the soil HMs under different land use are presented in Table S7. The mean contents of Zn and Cr were significantly higher than the others, while the mean contents of Cd and Hg were the lowest (Figure 2). For different land use types, the mean concentrations (mg/kg) for Cu raged from 27.24 to 31.29, for Cr from 86.71 to 90.10, for Ni from 32.39 to 35.85, for Zn from 87.23 to 95.44, for Pb from 27.56 to 36.43, for Cd from 0.200 to 0.227, for As from 8.20 to 10.42, and for Hg from 0.063 to 0.106, respectively (Table S7). The mean content of most HMs under different land use types have no significant difference, which is also consistent with the low level of soil HM pollution as a whole. However, the Hg concentrations in construction land were significantly higher than that in other three land use types. In general, there are two influencing factors of Hg pollution, long distance transport and local emissions. The Hg pollution via long distance transport caused by coal burning as a for heating system in winter in north China may increase the Hg concentrations in all land use types in Chongming Island at a relatively equal level [34,35]. Therefore, the local emissions such as the emission of coal-fired power plants and fossil fuel combustion may contribute to the difference of Hg pollution between construction land and the other three land use types [36]. 

Based on the background value in China [25], the exceeding rates for all HMs were calculated and the results indicated that most HMs (except As in construction land) in four land types exceeded their corresponding background value (Table S8). In addition, the results also demonstrated that the average exceeding rate for all HMs in the construction land was higher than that in other three land use types, and the order of the mean exceeding rate was as follows: construction land (78.13%) > wetland (71.32%) > agricultural land (71.08%) > forest land (70.08%). Obviously, most of the soils in the study area were enriched to some extent by soil HMs, which may be attributed to anthropogenic sources or natural sources. Generally, the HM pollution for construction and agricultural land is more susceptible to external human activities than wetland and forest land, such as industrial production, the combustion of fossil fuels, and the use of pesticides and fertilizers [1].

### 3.3. Ecological Risk of Soil HMs under Different Land Use

**Figure 3:**
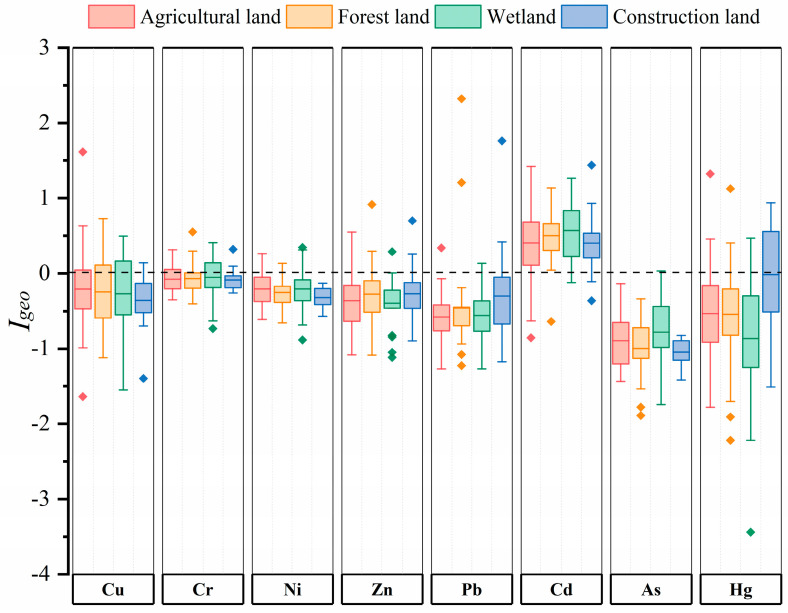
$I_{geo}$ values for the HMs in Chongming Island from the different land uses.

The *I_geo_* and *RI* were applied to appraise the risk level caused by the soil HM pollutions in the study area. The mean *I_geo_* values of soil HMs had a decreasing order of Cd (0.457) > Cr (−0.071) > Ni (−0.235) > Cu (−0.248) > Zn (−0.329) > Pb (−0.503) > Hg (−0.512) > As (−0.926) (Figure 3). Thus, the dominant contaminant metal in the soils was Cd with the level of “unpolluted to moderately polluted”, and followed by Cr, Ni, Cu, Zn, Pb, Hg and As with the level of “unpolluted”, indicating that the soils of the study area were slightly polluted by soil HMs except Cd. The *I_geo_* values of Cr, Cd and As had a peak in the wetland, while the *I_geo_* values of Cu and Ni were the highest in the agricultural land. The *I_geo_* values for Pb and Hg in construction land were higher than the other land uses, but the Zn reached its high values in forest land. Generally, the *I_geo_* values of all HMs except Cd in four land use types were smaller than zero, which suggests that there was no contamination posed by these HMs in the study area.

**Figure 4:**
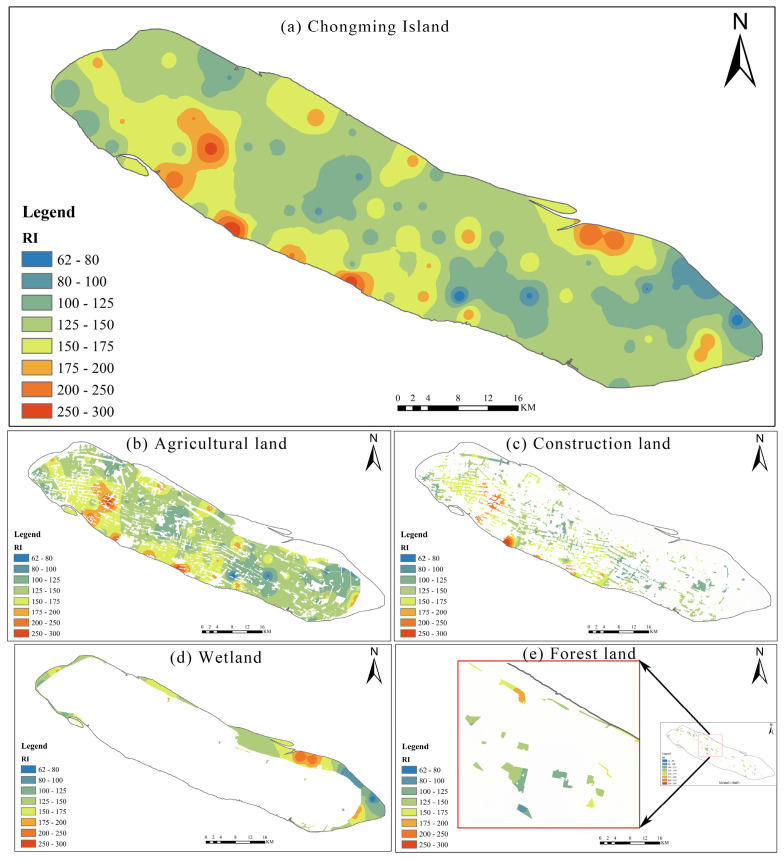
Distribution map of the potential ecological risk index (RI) in the soils of the study area: (**a**) Chongming Island, (**b**) agricultural land, (**c**) construction land, (**d**) wetland, and (**e**) forest land.

RI can reflect the level of the potential ecological risk caused by hazardous elements and represents the sensitivity of various biological communities to toxic substances (Zhang et al. 2019b). The mean $E_{r}^{i}$ values of soil HMs followed the order of: Cd (64.46) > Hg (47.10) > As (8.17) > Cu (6.69) > Ni (6.45) > Pb (5.76) > Cr (2.88) > Zn (1.23) (Figure S3). The average $E_{r}^{i}$ values for Cd and Hg were > 40, significantly higher than that of the other HMs, while the $E_{r}^{i}$ values for Cu, Cr, Ni, Zn, Pb and As were 2.41−23.01, 1.81−4.40, 4.07−9.54, 0.69−2.83, 3.12−37.50 and 4.05−15.36, respectively. According to the $E_{r}^{i}$ classifications of HMs (Table S2), the risk level for Cd and Hg was “Moderated potential risk”, while for Cu, Cr, Ni, Zn, Pb and As was “low potential risk”. According to the classification criteria of RI (Table S2), the RI values ranged from 62.56 to 299.62 (Table S9), meaning that the overall ecological risk of soil HMs was between the level of “Low ecological risk” and “Moderate ecological risk”. Results also showed that the average RI values for four land uses were as follows: construction land (157.51) > wetland (141.08) > forest land (139.01) > agricultural land (138.92). Land use was an important factor affecting the HM concentration and distribution in soils [16]. In this study, the environmental risk of HMs in the construction land were basically higher than those in other land use, which might be related long-term industrialization and rapid urbanization [1]. As shown in Figure 4a, the high ecological risk area was principally distributed in the central and western regions of the study area. The main land use types in the western region of the study area are construction land and agricultural land, which is the area nearest to the Shanghai metropolis and the most densely populated area. Therefore, the high RI value in these areas may be closely related to industrial and agricultural activities (e.g., waste emissions and the use of fertilizers and pesticides) [37], which can also be seen from Figure 4b, c. However, the southeastern area of the study area has a small population and the main land use type was wetland. Its high RI value may be due to the HM pollution caused by estuarine alluvium (Figure 4d) [38]. The environmental risk of HMs in the forest land was generally at a relatively low level (Figure 4e). The reason might be that the forest land is generally less influenced by human input and plants can reduce the content of some HMs in the soil [9,39,40].

### 3.4. Human Risk Assessment of HMs in Soils

According to WHO, the contamination caused by HM in food occurs mainly through the pollution of air, water and soil, threatening the health of people by the food chain. Chongming Island is the largest agricultural product supply base for Shanghai Metropolis, and it needs to be paid great attention because its soil HM pollution is closely related to human health risks. Therefore, the NCR and CR posed by soil HMs under different land uses for two age groups (children and adults) through different exposure routes in the study area were evaluated. According to the USEPA (2009), if the hazard quotient (HQ) values was < 1, the exposed individual is unlikely to be significantly affected by adverse health effects [27]. As illustrated in Table S10, the results showed that the HQ values for three exposure pathways in the four land uses were all within the safety threshold (< 1), suggesting that the harmful effects of soil HMs on human health are within acceptable ranges. Results also showed that the NCR of HMs through different exposure pathways in four land use types followed the order of HQ_ing_ > HQ_derm_ > HQ_inh_, whereas, compared with adults, there are significantly higher NCR in children (Table S10), which is in accordance with the previous reports (Chen et al. 2016; Zhou et al. 2019). A possible explanation is that children are more exposed to HMs in soils as their playing habits leading to the higher NCR in children than in adults.

**Figure 5:**
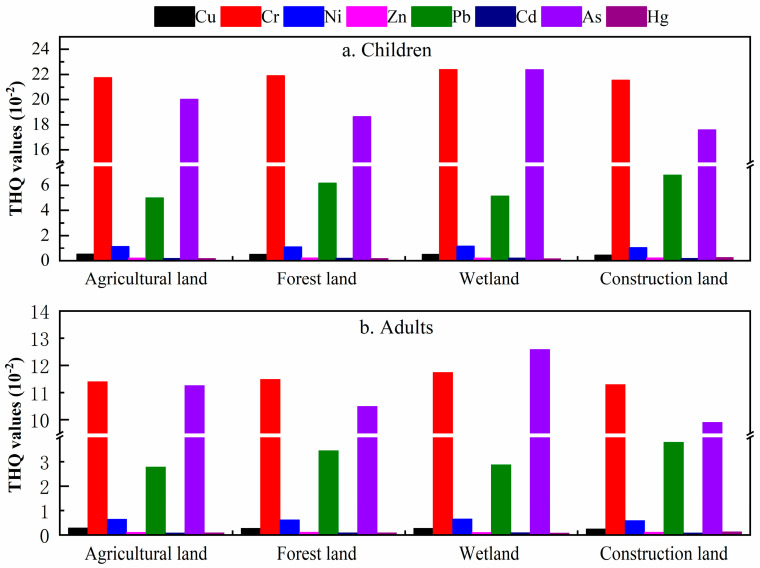
The non-carcinogenic risk (total hazard quotient (THQ)) under the different land uses for: (**a**) children and (**b**) adults.

According to USEPA (2009), the total hazard quotient (THQ) posed by the exposure to more than one HM in four land use types were determined. The results indicated that all the THQ values were <1, indicating that there was no adverse NCR caused by HMs in all the land use types. The performance of the THQ value was closely relevant to the type of land use. As illustrated in Figure 5, the order of THQ values for both children and adults in agricultural land and forest land was Cr > As > Pb > Ni > Cu > Zn > Cd > Hg, while in construction land it followed the order of Cr > As > Pb > Ni > Cu > Hg > Zn > Cd. The trend for the THQ rank order for adults in wetland was As > Cr > Pb > Ni > Cu > Zn > Cd > Hg, while for children it was As ≈ Cr > Pb > Ni > Cu > Zn > Cd > Hg. The results of THQ also indicated that the higher risk was mainly posed by Cr and As in all four land use types. The THQ values of Cr in for the four land use types followed the order: wetland > agricultural land > forest land > construction land, and the order for Cr is: wetland > forest land > agricultural land > construction land. It is worth mentioning that the metal of Cr and As have the highest risk values in wetland. The reason for this is not clear but it may be related to the accumulation of pollutants transported by rivers [41].

As shown in Table 2, the CR posed by Cr, Ni, Pb, As and Cd were assessed. The TCR values of As ranged from 1.53 × 10^−5^ to 4.52 × 10^−5^ meaning that the CR for these HMs was acceptable (1 × 10^−6^ < TCR < 1 × 10^−4^). Moreover, the TCR values for Cr, Ni, Pb and Cd ranged from 2.49×10^−7^ to 5.72×10^−7^, 1.86 × 10^−9^ to 4.55 × 10^−9^, 2.81 × 10^−7^ to 8.48 × 10^−7^, and 8.61 × 10^−11^ to 2.16 × 10^−10^, respectively, demonstrating that the carcinogenic risk of these HMs was negligible because their TCR values were < 1 × 10^−6^. Moreover, The CR value for the different population followed the order of children > adults, and the high CR value of children may be related to their playing habits, higher intake rate of HMs and lower status [42]. In a word, it can be concluded that Cr and As are the major elements that posed a higher NCR to the human population, while As showed a higher CR to both adults and children.

### 3.5. Source Apportionment of HMs

**Figure 6:**
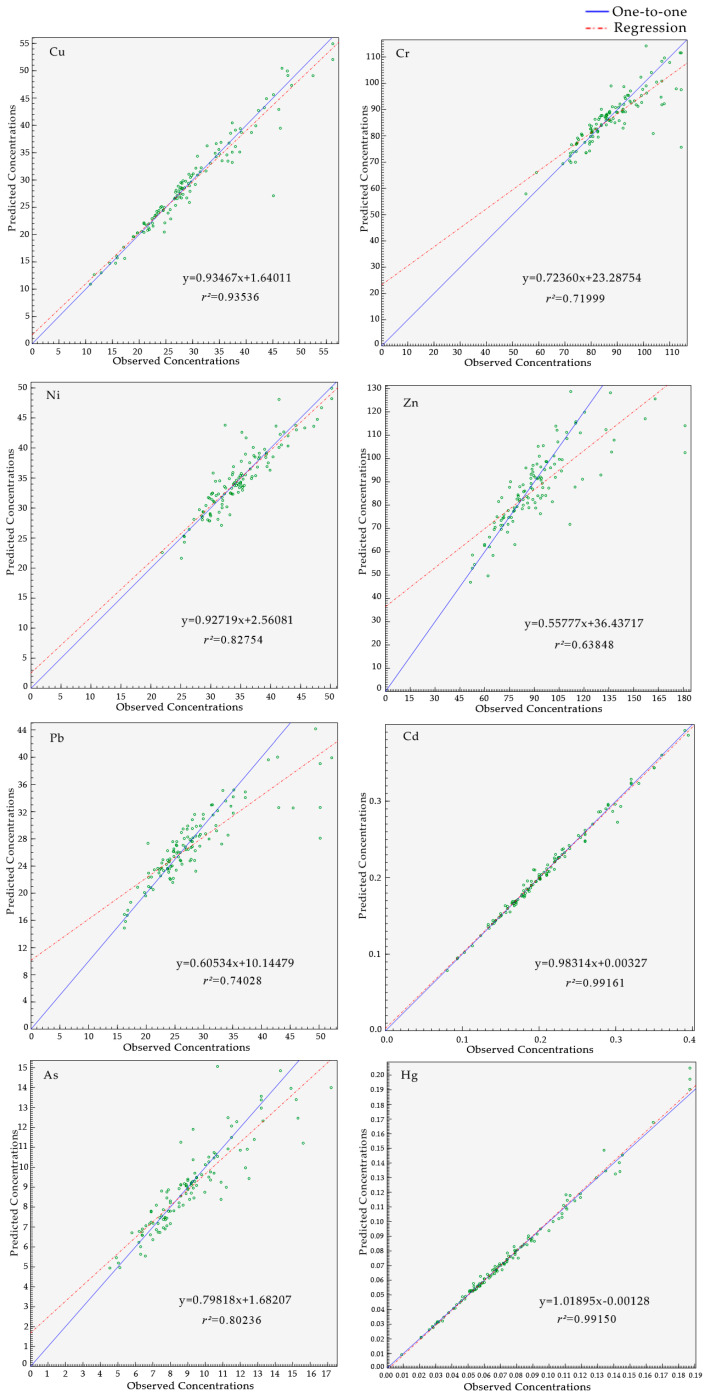
Fitting between the observed and the predicted concentrations by the positive matrix factorization (PMF) model.

The PMF model was applied to recognize the sources and further quantify the source contributions of the soil HMs in this study. During the analysis of the PMF model, there is no definite number of factors, which may lead to some errors. When the number of factors is too large, one source may be decomposed into multiple sources; instead, different pollution sources may also be merged into one single source. Therefore, the reasonable number of factors should be determined by finding the minimum and stable Q value and controlling the residual matrix E. In this study, the number of factors was set to 2–6, and the number of runs was 20. According the PMF processing results, when the factor number was set to 4, the value of Q was minimum and stable, the most residual was within −3 and 3, and the signal-to-noise (S/N) ratio of all HMs exceeded 9. Moreover, the fitting coefficient between the measured value and the predicted value (*r^2^*) of all HMs was 0.935 (Cu), 0.720 (Cr), 0.828 (Ni), 0.638 (Zn), 0.740 (Pb), 0.992 (Cd), 0.802 (As), and 0.992 (Hg), respectively (Figure 6). The *r^2^* values were > 0.6, which indicates that the PMF model is reasonable and the results are reliable.

**Figure 7:**
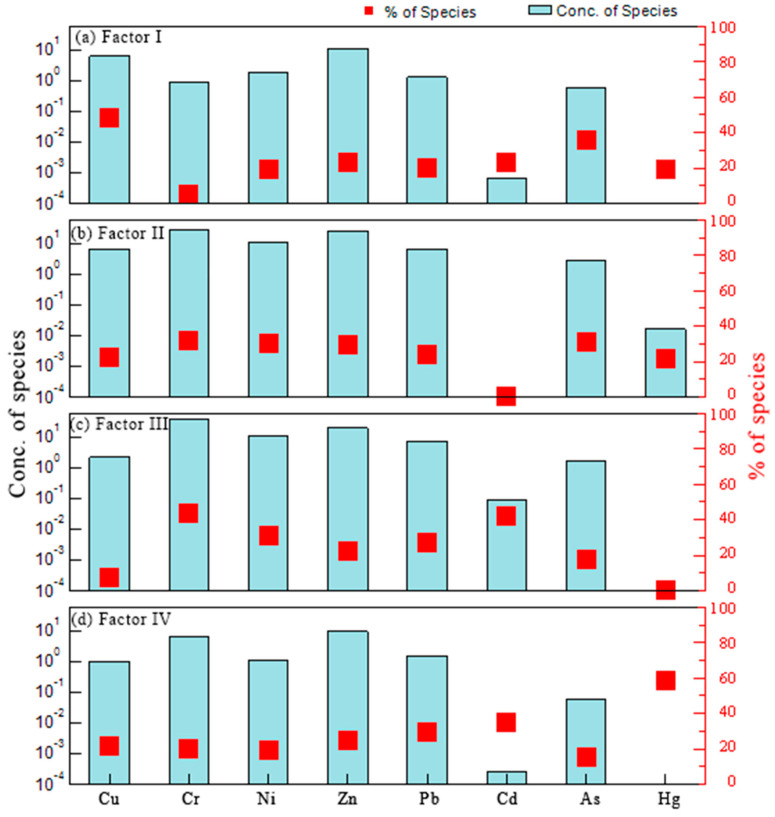
Source profiles and the source contributions of the HMs from the PMF model ((**a** )Factor I, (**b**) Factor II, (**c** )Factor III, and (**d**) Factor IV).

**Figure 8:**
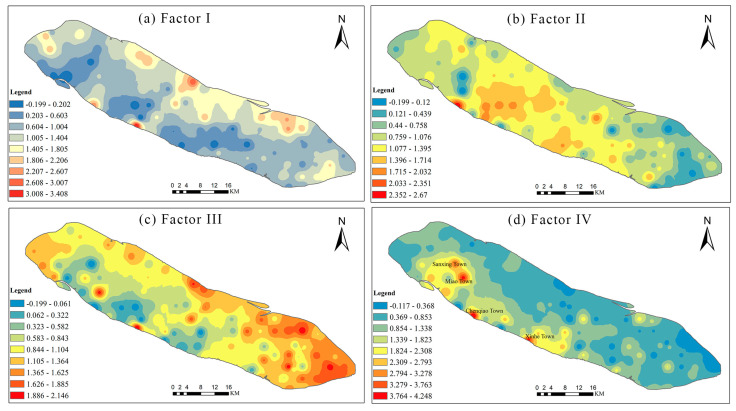
Spatial distribution map of the contribution rates of the four factors based on GIS ((**a**) Factor I,(**b**) Factor II, (**c**) Factor III, and **(d)** Factor IV).

As illustrated in Figure 7, the PMF analysis showed that there were four factors, named Factor I, Factor II, Factor III, and Factor IV, respectively, affecting the distribution, concentration and accumulation of HMs in the study area. In particular, for more accurately recognizing potential sources of soil HMs and potential pollution hotspots, the spatial distribution of four factors were visualized by using the spatial technology of geo-statistic (Figure 8).

Factor I accounted for 24.25% of the contribution rate, was mostly loaded on Cu (48.43%), Zn (23.44%), and As (35.88%) (Figure 7a). The mean contents of Cu, Zn, and As exceeded their reference background values, suggesting that these HMs were probably caused by human activities. There were many previous researches which presented that the accumulation of these elements is relevant to the application of pesticides or fertilizer (Table S11). Cai et al. (2019) reported that the use efficiency of pesticides and fertilizers in the agricultural production in China were lower, and that 70% of them flow into other media (soil, water, or air) [36]. Yang et al. (2017) demonstrated that Cu, Zn and As are closely related to the fertilizer and pesticide application in agricultural production [43]. Liang et al. (2017) and Lin et al. (2018) showed that Cu, Zn and As were usually considered to derive from the application of agricultural fertilizer and pesticides [12,44]. Agricultural land is the dominant way of land use in Chongming Island, and agriculture is the leading industry, which leads to a large amount of pesticides and fertilizers [37]. Furthermore, the concentrations of Cu, Zn, and As had similar spatial distribution trends, and the high value regions of these HMs were mostly distributed in agricultural land (Figure S2). Therefore, Factor I originated from an agricultural source.

Factor II accounted for 24.25% of the contribution rate, was mostly loaded on Ni (29.96%) and Zn (29.59%) (Figure 7b). Ni and Zn were generally considered to originate from soil parent materials. The parent material in Chongming Island is mainly river alluvium. The contents of Ni and Zn were evenly distributed in space in the study area (Figure S2), and were close to the background values in the soils. This spatial distribution is consistent with the results of the environmental risk of Ni and Zn. Hence, Factor II was identified as a natural source factor.

Factor III made up 23.84 % of the total contribution. Then, the metal of Cr, Ni, and Cd were the main loading HMs and accounted for 43.46%, 31.11%, and 42.23%, respectively (Figure 7c). Previous studies reported that these HMs would derive from industrial production, such as electroplating, battery production, mining, and metal manufacturing (Table S11). The mean concentrations of Cr, Ni, and Cd basically exceeded their background values (Table 2). The spatial distribution of the Cr, Ni, and Cd concentrations had a similar trend, and their hot spots were bound up with the industrial distribution, such as metal product, ship building, light industry and chemical industry (Figure S2). In particular, because the study area is an estuary alluvial island, the accumulation of some HMs (e.g., Cr, Cd, Ni) may also be transported by rivers and then deposited. Sun et al. (2018) found that the HM content in the sediments adjacent to Chongming Dongtan wetland was significantly higher than that in the other areas of the Yangtze River estuary, indicating that the metal content was affected by sediment deposition [45]. Zheng et al. (2016) also showed that fluvial transport is an important factor affecting the HM content in Chongming Island wetland [38]. The spatial distribution of Factor III indicated that the high value regions of soil HMs (Cr, Ni, and Cd) are primarily concentrated in wetlands and construction land (Figure 8c). It can be inferred that industrial activities and river transportation are the primary factors influencing the enrichment of these metals in accordance with the results by Zheng et al. (2016) [38]. Thus, Factor III may represent the source of industrial activity and river transportation.

Factor IV which accounted for 28.12% of the total contribution rate was defined by Hg (59.03%) and Pb (29.77 %) (Figure 7d). Pb is a representative element of transportation emissions [44,46]. Furthermore, the discharge of combustion is an significant source of Pb in atmosphere deposition [47]. Hg is also associated with the burning of fossil fuels [36]. These elements may get into soils through atmospheric deposition as reported by Lin et al. (2018) [12]. The high value of Pb and Hg (Factor IV) was mainly concentrated in the areas with heavy traffic and industrial emissions, such as Xinhe Town, Chenqiao Town, Sanxing Town and Miao Town (Figure 8d). Therefore, Factor IV can be considered as the atmosphere deposition.

**Figure 9:**
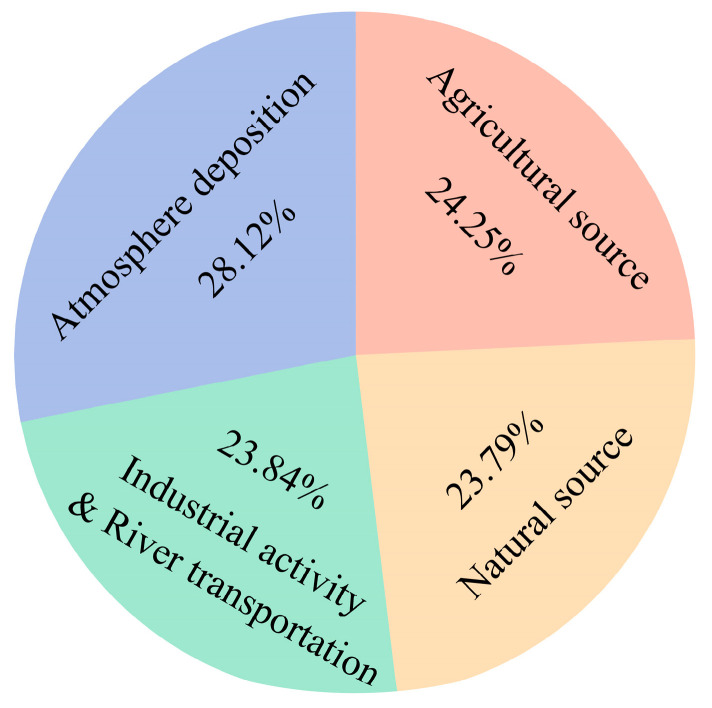
Pollution source apportionment of the soil HMs.

In summary, atmosphere deposition was apportioned as the largest percent contribution (28.12%) for the soil HMs in Chongming Island, followed by agricultural source (24.25%), industrial activity and river transportation (23.84%), and natural source (23.79%) (Figure 9). It is apparent that anthropogenic sources are the predominant factors influencing the contents of soil HMs in Chongming Island, especially through atmosphere deposition and agricultural activities. The following measures can be taken to control pollution: (1) optimizing the structure and increasing the prevention and control of key industries; (2) implementing the strict supervision of heavy metal pollution sources; (3) strengthening the construction of heavy metal environment supervision. In addition, more attention should be paid to solve the problem of agricultural pollution as agriculture is one of the main development economic activities of Chongming Island in the future. To prevent and control agricultural pollution, there is an urgent need for the establishment of standards for sewage discharge and the optimization of fertilization to guarantee soil quality and the safety of agricultural products. Meanwhile, regular monitoring and an integrated environmental management system to cover the whole agricultural production chain should be gradually established.

## 4. Conclusions

In this study, based on a detailed survey of soil HM concentrations, the environmental risk and source allocation of soil HMs were determined. The mean content of soil HMs (except As) was higher than the corresponding background value in China, but were lower than that in the urban zone in Shanghai. The average content of most HMs under different land use had no significantly difference, and the Hg content in the construction land was significantly higher than that in other three land uses. The evaluation results of *I_geo_* and RI indicated that the soil HM pollution was “unpolluted” or “unpolluted to moderately polluted”, and the risk level was “low potential risk” or “moderated potential risk”. Due to the lower population density and urbanization level, human activities have less impact on the study area, resulting in lower levels of soil HM pollution and human risk. The spatial distribution of *RI* showed that the high ecological risk areas were primarily distributed in the central and western region in Chongming Island, which were associated with industrial production, agricultural activities and estuarine deposition. The PMF model coupled with GIS-spatial analysis was applied to analyze and quantify the potential sources of soil HM pollution, and the results showed that agricultural activities, natural source, industrial discharge and river transportation, and atmosphere deposition were the main determining factors for the level of soil HMs in Chongming Island. Although the results of the HRA indicated that the NCR and CR caused by the soil HMs were insignificant, the protection and management of the soil environmental quality related to HMs still needs to be paid great attention because the study area is the largest agricultural product supply base for Shanghai Metropolis.

## Figures and Tables

**Table 1:** The soil heavy metal (HM) concentrations (mg/kg) in the study area.

Location		Cu	Cr	Ni	Zn	Pb	Cd	As	Hg	Reference
Chongming Island	Mea	30.22	87.96	34.68	95.60	29.95	0.208	9.14	0.077	This study
	Min	10.90	55.10	21.90	51.40	16.20	0.080	4.54	0.009	
	Max	104.00	134.20	51.30	210.00	195.00	0.394	17.20	0.244	
	CV	37.2%	14.0%	15.6%	26.9%	66.8%	29.5%	26.7%	48.9%	
Urban Zone in Shanghai		59.25	107.9	31.14	301.4	70.69	0.52	NA	NA	[21]
Background in China ^a^		22.6	61.0	26.9	74.2	26.0	0.097	11.2	0.065	[25]
Standard Level ^b^		100	250	190	300	170	0.6	20	1.0	(GB 15618–2018)
UCC ^c^		14.3	35	18.6	52	17	0.102	2	0.056	[33]
World Background ^d^		30	70	40	90	35	0.35	7.2	0.06	[1]

CV: Coefficient of Variation. ^a^ Soil background values of HMs in China. ^b^ Standard level: According to Soil Environmental Quality Management Standard in China (GB 15618–2018), it indicates the negligible risk for the quality and safety of agricultural products, crop growth or soil ecological environment if the standard level was met. ^c^ UCC: upper continent crust. ^d^ Background values of HMs for world soils.

**Table 2:** Total carcinogenic risk (TCR) of the HMs for children and adults in the different land use.

HMs	Agricultural Land		Forest Land		Wetland		Construction Land	
	Children	Adults	Children	Adults	Children	Adults	Children	Adults
Cr	5.55 × 10^−7^ *	2.51 × 10^−7^ *	5.59 × 10^−7^ *	2.53 × 10^−7^ *	5.72 × 10^−7^ *	2.58 × 10^−7^ *	5.50 × 10^−7^ *	2.49 × 10^−7^ *
Ni	4.49 × 10^−9^ *	2.03 × 10^−9^ *	4.34 × 10^−9^ *	1.96 × 10^−9^ *	4.55 × 10^−9^ *	2.06 × 10^−9^ *	4.11 × 10^−9^ *	1.86 × 10^−9^ *
Pb	6.21 × 10^−7^ *	2.81 × 10^−7^ *	7.71 × 10^−7^ *	3.48 × 10^−7^ *	6.42 × 10^−7^ *	2.90 × 10^−7^ *	8.48 × 10^−7^ *	3.83 × 10^−7^ *
Cd	1.93 × 10^−10^ *	8.71 × 10^−11^ *	2.02 × 10^−10^ *	9.14 × 10^−11^ *	2.16 × 10^−10^ *	9.75 × 10^−11^*	1.91 × 10^−10^*	8.61 × 10^−11^ *
As	3.86 × 10^−5^ **	1.74 × 10^−5^ **	3.60 × 10^−5^ **	1.62 × 10^−5^ **	4.32 × 10^−5^ **	1.94 × 10^−5^ **	3.40 × 10^−5^ **	1.53 × 10^−5^ **

* Indicates a negligible risk to human health (TCR < 1 × 10^−6^). ** Indicates an acceptable risk to human health (1 × 10^−6^ < TCR < 1 × 10^−4^).

## Supplementary Materials

The following are available online at https://www.mdpi.com/1660-4601/17/13/4841/s1. Figure S1: Comparison between Shanghai City and Chongming Island: (1) The proportion of land use; (2) The proportion of the first, second and third industries; (3) a. Density of population, b. urbanization rate, c. GDP per unit land. Figure S2: Distribution map of the average concentration of heavy metals (Cu, Cr, Ni, Zn, Pb, Cd, As and Hg) in Chongming Island. Figure S3: Distribution map of *E_r_^i^* values of heavy metals (Cu, Cr, Ni, Zn, Pb, Cd, As and Hg) in Chongming Island. Table S1: Classifications of the HMs according to the geo-accumulation index. Table S2: Grades of the HMs according to the $E_{r}^{i}$ and RI. Table S3: Definition and reference value of some parameters for the human health risk assessment of HMs in the soil. Table S4: Parameter values of RfD and SF in the assessment model of health risk. Table S5: The physicochemical parameter of soils in Chongming Island. Table S6: Classifications of the coefficients of variation (CV). Table S7: Concentrations of the soil HMs in the study area under land use. Table S8: The exceeding rate for soil HMs under different land use (%). Table S9: The calculated *E_r_^i^* and RI in Chongming Island. Table S10: The hazard quotient of the HMs for three exposure pathways for children and adults in four land use types. Table S11: Major sources of HMs.
