# Supplementary material for: Risk Assessment and Source Apportionment of Soil Heavy Metals under Different Land Use in a Typical Estuary Alluvial Island
Source: Int J Environ Res Public Health. 2020 Jul 5;17(13):4841. doi: 10.3390/ijerph17134841 (PMC7369809; doi:10.3390/ijerph17134841)
Supplement: Supplementary file 1 [file ijerph-17-04841-s001.pdf]

International Journal of
Environmental Research and Public Health

MDPI

Supplementary materials

Table S1. Classifications of the HMs according to the geo-accumulation index.

Classes	Values	Description
0	$I_{geo} \leq 0$	Unpolluted
1	$0 < I_{geo} \leq 1$	From unpolluted to moderately polluted
2	$1 < I_{geo} \leq 2$	Moderately polluted
3	$2 < I_{geo} \leq 3$	From moderately polluted to strongly polluted
4	$3 < I_{geo} \leq 4$	Strongly polluted
5	$4 < I_{geo} \leq 5$	From strongly polluted to extremely polluted
6	$I_{geo} > 5$	Extremely polluted

Table S2. Grades of the HMs according to the E_r^i and RI

E_r^i	Risk Level	RI	Risk Level
$E_r^i < 40$	Low potential risk	RI < 150	Low ecological risk
$40 \leq E_r^i < 80$	Moderate potential risk	150 ≤ RI < 300	Moderate ecological risk
$80 \leq E_r^i < 160$	Considerable potential risk	300 ≤ RI< 600	High ecological risk
$160 \leq E_r^i < 320$	High potential risk	600 ≤ RI	Significantly high ecological risk
$320 \leq E_r^i$	Serious		

Table S3. Definition and reference value of some parameters for human health risk assessment of HMs in soil.

Parameter	Definition	Reference Values		Reference
		Adult	Children	
IngR	The ingestion frequency (mg/day)	200	100	[1]
InhR	The inhalation frequency/(m^3/day)	15	7.5	[2]
PEF	The particle emission factor/(m^3/kg)	1.36×10^9	1.36×10^9	[3]
SA	Surface area of exposed skin/(cm^2)	4350	1600	[2]
AF	Adhesiveness degree of skin/(mg/(cm^2 day))	0.07	0.2	[4]
BW	Body weight/(kg)	53.1	15	[5]
ED	Exposure duration/(year)	Non-carcinogenic 24 carcinogenic 24	Non-carcinogenic 6 carcinogenic 30	[5]
ABS	Absorption factor of skin	0.001	0.001	[4]
EF	The exposure frequency/(day/year)	350	350	[4]
CF	The conversion factor/(kg/mg)	1×10^{-6}	1×10^{-6}	[6]
AT	The average exposure time/(day)	Non-carcinogenic ED × 365 = 8760; carcinogenic 365 × 70 = 25550	Non-carcinogenic ED × 365 = 2190; carcinogenic 365 × 70 = 25550	[5]

Table S4. Parameter values of *RfD* and *SF* in the assessment model of health risk.

Parameter	Cu	Cr	Ni	Zn	Pb	Cd	As	Hg
RfD_{ing}	4.00×10^{-2}	3.00×10^{-3}	2.00×10^{-2}	3.00×10^{-1}	3.50×10^{-3}	1.00×10^{-3}	3.00×10^{-4}	3.00×10^{-4}
RfD_{der}	1.20×10^{-2}	6.00×10^{-5}	5.40×10^{-3}	6.00×10^{-2}	5.25×10^{-4}	1.00×10^{-5}	1.23×10^{-4}	2.4×10^{-5}
RfD_{inh}	4.02×10^{-2}	2.86×10^{-5}	2.06×10^{-2}	3.00×10^{-1}	3.52×10^{-3}	1.00×10^{-5}	3.00×10^{-4}	3.00×10^{-4}
SF_{ing}					8.50×10^{-3}		1.50	
SF_{der}							3.66	
SF_{inh}		4.20 × 10	8.40×10^{-1}			6.3	1.51 × 10	

Table S5. The physicochemical parameter of soils in Chongming Island.

Unit	Statistic	pH	CEC	TP	TN	OC
		-	cmol (+)/kg	mg/kg	g/kg	g/kg
Agricultural Land	Mean	8.26	12.25	814.92	1.04	11.25
	Min	7.63	4.43	455.00	0.35	4.50
	Max	8.67	26.60	1460.00	1.83	26.10
	SD	0.25	3.97	176.73	0.34	3.80
	Skewness	-0.72	0.91	0.64	0.37	1.41
	CV (%)	3.03	32.46	21.69	32.64	33.78
Forest Land	Mean	8.23	12.94	716.45	1.08	13.03
	Min	7.90	5.72	381.00	0.16	5.44
	Max	8.73	23.30	1020.00	1.94	31.20
	SD	0.20	4.76	138.18	0.39	5.10
	Skewness	0.44	0.65	-0.12	0.10	1.32
	CV (%)	2.45	36.80	19.29	35.76	39.15
Wetland	Mean	8.49	12.01	765.76	0.83	9.53
	Min	7.78	3.33	545.00	0.19	2.10
	Max	10.09	25.10	1290.00	1.30	14.40
	SD	0.46	5.98	186.99	0.34	4.00
	Skewness	2.24	0.55	1.48	–0.50	-0.81
	CV (%)	5.46	49.79	24.42	40.43	41.96
Construction Land	Mean	8.20	11.08	866.13	0.95	10.50
	Min	7.94	5.67	536.00	0.57	4.80
	Max	8.46	18.70	1510.00	1.51	17.10
	SD	0.15	3.38	241.47	0.26	3.27
	Skewness	–0.01	0.57	1.18	0.81	0.54
	CV (%)	1.88	30.48	27.88	27.82	31.13
The Study Area	Mean	8.28	12.25	787.01	1.01	11.40
	Min	7.63	3.33	381.00	0.16	2.10
	Max	10.09	26.60	1510.00	1.94	31.20
	SD	0.29	4.51	186.33	0.35	4.33
	Skewness	1.89	0.72	0.99	0.22	1.14
	CV (%)	3.45	36.84	23.68	35.06	38.03

SD: Standard Deviation CV: Coefficient of Variation.

Table S6. Classifications of coefficients of variation (CV).

Classes	Values	Description
1	CV < 20%	Low variability
2	20% < CV ≤ 50%	Moderate variability
3	50% < CV ≤ 100%	High variability
4	CV > 100%	Exceptionally high variability

Table S7. Concentrations of soil HMs in the study area under land use.

Unit	Statistic	Cu	Cr	Ni	Zn	Pb	Cd	As	Hg
		mg/kg	mg/kg	mg/kg	mg/kg	mg/kg	mg/kg	mg/kg	mg/kg
Agricultural Land	Mea	31.29	87.52	35.35	89.12	26.67	0.202	9.32	0.074
	Min	10.90	71.80	26.40	52.60	16.20	0.080	6.22	0.028
	Max	104.00	114.00	48.50	163.00	49.40	0.390	15.30	0.244
	SD	13.37	11.28	5.25	22.31	5.61	0.062	2.32	0.035
	CV (%)	0.43	0.13	0.15	0.25	0.21	0.309	0.25	0.472
Forest Land	Mean	30.03	88.14	34.16	95.08	33.10	0.212	8.68	0.074
	Min	15.60	69.20	25.60	52.50	16.70	0.093	4.54	0.021
	Max	56.20	134.20	44.30	210.00	195.00	0.320	13.30	0.213
	SD	9.47	12.16	4.61	27.28	30.92	0.052	2.04	0.034
	CV (%)	0.32	0.14	0.14	0.29	0.93	0.243	0.23	0.466
Wetland	Mean	30.21	90.10	35.85	87.23	27.56	0.227	10.42	0.063
	Min	11.60	55.10	21.90	51.40	16.20	0.134	5.03	0.009
	Max	47.80	121.60	51.30	136.00	42.80	0.350	17.20	0.135
	SD	10.45	17.03	7.87	20.72	7.50	0.070	3.61	0.031
	CV (%)	0.35	0.19	0.22	0.24	0.27	0.311	0.35	0.493
Construction Land	Mean	27.24	86.71	32.39	95.44	36.43	0.200	8.20	0.106
	Min	12.90	76.50	27.20	59.90	17.30	0.080	6.30	0.034
	Max	37.40	114.40	36.90	181.10	132.30	0.394	9.50	0.187
	SD	6.24	9.01	2.64	27.32	26.34	0.062	0.94	0.043
	CV (%)	0.23	0.10	0.08	0.29	0.72	0.309	0.11	0.403

SD: Standard Deviation CV: Coefficient of Variation.

Table S8. The exceeding rate for soil HMs under different land use (%).

	Cu	Cr	Ni	Zn	Pb	Cd	As	Hg	Mean
Agricultural Land	82.35	100.00	98.04	70.59	45.10	96.08	21.57	54.90	71.08
Forest Land	72.73	100.00	93.94	84.85	48.48	96.97	9.09	54.55	70.08
Wetland	76.47	88.24	82.35	76.47	58.82	100.00	35.29	52.94	71.32
Construction Land	81.25	100.00	100.00	87.50	68.75	100.00	0.00	87.50	78.13

Table S9. The calculated E_r^i and RI in Chongming Island.

	E_r^i (Individual Metal)								RI (Multi-metal)
	Cu	Cr	Ni	Zn	Pb	Cd	As	Hg	
Mean	6.69	2.88	6.45	1.23	5.76	64.46	8.16	47.10	142.73
Min	2.41	1.81	4.07	0.69	3.12	24.89	4.05	5.54	62.56
Max	23.01	4.40	9.54	2.83	37.50	121.99	15.36	150.15	299.62

Table S10. The hazard quotient of HMs for three exposure pathways for children and adult in four land use types.

Risk	Gender	Land Use	Cu	Cr	Ni	Zn	Pb	Cd	As	Hg
HQ_{ing}	Children	Agricultural land	5.00×10^{-3}	1.86×10^{-1}	1.13×10^{-2}	1.90×10^{-3}	4.87×10^{-2}	1.29×10^{-3}	1.99×10^{-1}	1.57×10^{-3}
		Forest land	4.80×10^{-3}	1.88×10^{-1}	1.09×10^{-2}	2.03×10^{-3}	6.05×10^{-2}	1.36×10^{-3}	1.85×10^{-1}	1.57×10^{-3}
		Wetland	4.83×10^{-3}	1.92×10^{-1}	1.15×10^{-2}	1.86×10^{-3}	5.03×10^{-2}	1.45×10^{-3}	2.22×10^{-1}	1.35×10^{-3}
		Construction land	4.35×10^{-3}	1.85×10^{-1}	1.04×10^{-2}	2.03×10^{-3}	6.65×10^{-2}	1.28×10^{-3}	1.75×10^{-1}	2.25×10^{-3}
	Adults	Agricultural land	2.83×10^{-3}	1.05×10^{-1}	6.38×10^{-3}	1.07×10^{-3}	2.75×10^{-2}	7.31×10^{-3}	1.12×10^{-1}	8.87×10^{-4}
		Forest land	2.71×10^{-3}	1.06×10^{-1}	6.17×10^{-3}	1.14×10^{-3}	3.42×10^{-2}	7.67×10^{-4}	1.04×10^{-1}	8.87×10^{-4}

		Wetland	2.73×10^{-3}	1.08×10^{-1}	6.47×10^{-3}	1.05×10^{-3}	2.84×10^{-2}	8.18×10^{-4}	1.25×10^{-1}	7.62×10^{-4}
		Construction land	2.46×10^{-3}	1.04×10^{-1}	5.85×10^{-3}	1.15×10^{-3}	3.76×10^{-2}	7.23×10^{-4}	9.87×10^{-2}	1.27×10^{-3}
HQ_{inh}	Children	Agricultural land	2.74×10^{-7}	1.08×10^{-3}	6.05×10^{-7}	1.05×10^{-7}	2.67×10^{-6}	7.13×10^{-6}	1.09×10^{-5}	8.66×10^{-8}
		Forest land	2.63×10^{-7}	1.09×10^{-3}	5.85×10^{-7}	1.12×10^{-7}	3.31×10^{-6}	7.49×10^{-6}	1.02×10^{-5}	8.65×10^{-8}
		Wetland	2.65×10^{-7}	1.11×10^{-3}	6.13×10^{-7}	1.03×10^{-7}	2.76×10^{-6}	7.99×10^{-6}	1.22×10^{-5}	7.44×10^{-8}
		Construction land	2.39×10^{-7}	1.07×10^{-3}	5.54×10^{-7}	1.12×10^{-7}	3.65×10^{-6}	7.06×10^{-6}	9.63×10^{-6}	1.24×10^{-7}
	Adults	Agricultural land	1.55×10^{-7}	6.10×10^{-4}	3.42×10^{-7}	5.92×10^{-8}	1.51×10^{-6}	4.03×10^{-6}	6.19×10^{-6}	4.89×10^{-8}
		Forest land	1.49×10^{-7}	6.14×10^{-4}	3.30×10^{-7}	6.31×10^{-8}	1.87×10^{-6}	4.23×10^{-6}	5.76×10^{-6}	4.89×10^{-8}
		Wetland	1.50×10^{-7}	6.27×10^{-4}	3.47×10^{-7}	5.79×10^{-8}	1.56×10^{-6}	4.51×10^{-6}	6.92×10^{-6}	4.20×10^{-8}
		Construction land	1.35E-07	6.04×10^{-4}	3.13×10^{-7}	6.34×10^{-8}	2.06×10^{-6}	3.99×10^{-6}	5.44×10^{-6}	7.02×10^{-8}
HQ_{derm}	Children	Agricultural land	5.33×10^{-5}	2.98×10^{-2}	1.34×10^{-4}	3.04×10^{-5}	1.04×10^{-3}	4.14×10^{-4}	1.55×10^{-3}	6.28×10^{-5}
		Forest land	5.12×10^{-5}	3.00×10^{-2}	1.29×10^{-4}	3.24×10^{-5}	1.29×10^{-3}	4.35×10^{-4}	1.44×10^{-3}	6.28×10^{-5}
		Wetland	5.15×10^{-5}	3.07×10^{-2}	1.36×10^{-4}	2.97×10^{-5}	1.07×10^{-3}	4.63×10^{-4}	1.73×10^{-3}	5.39×10^{-5}
		Construction land	4.64×10^{-5}	2.96×10^{-2}	1.23×10^{-4}	3.25×10^{-5}	1.42×10^{-3}	4.10×10^{-4}	1.36×10^{-3}	9.01×10^{-5}
	Adults	Agricultural land	1.43×10^{-5}	8.02×10^{-3}	3.60×10^{-5}	8.17×10^{-6}	2.79×10^{-4}	1.11×10^{-4}	4.17×10^{-4}	1.69×10^{-5}
		Forest land	1.38×10^{-5}	8.08×10^{-3}	3.48×10^{-5}	8.71×10^{-6}	3.47×10^{-4}	1.17×10^{-4}	3.88×10^{-4}	1.69×10^{-5}
		Wetland	1.38×10^{-5}	8.26×10^{-3}	3.65×10^{-5}	7.99×10^{-6}	2.89×10^{-4}	1.25×10^{-4}	4.66×10^{-4}	1.45×10^{-5}
		Construction land	1.25×10^{-5}	7.95×10^{-3}	3.30×10^{-5}	8.75×10^{-6}	3.82×10^{-4}	1.10×10^{-4}	3.66×10^{-4}	2.42×10^{-5}

Table S11. Major sources of HMs.

HMs	Sources	References
Cu	Petrochemical wastewater	[7]
	Metal process and smelt	[8]
	Fertilizer and pesticides	[9–13]
Cr	Industrial activities	[14–18]
	Coal combustion	[8]
Ni	Industrial activities	[19]
Zn	Petrochemical wastewater	[7]
	Traffic	[20]
	Livestock manure	[21,22]
	Fertilizer and pesticides	[23,24]
Pb	Traffic emission	[8,25–28]
	Coal combustion	[29,30]
	Industrial waste	[30–32]
Cd	Lubricating oil and tires	[33]
	Coal combustion	[34]
	Fertilizer	[35,36]
	Galvanization	[32]
	Petrochemical	[37]
	Industrial activities	[38]

As	Coal combustion	[24,33,39]
	Industrial discharge	[40–42]
	Fertilizer and pesticides	[23,43]
Hg	Fertilizer	[25]
	Coal combustion	[27,30,44–47]

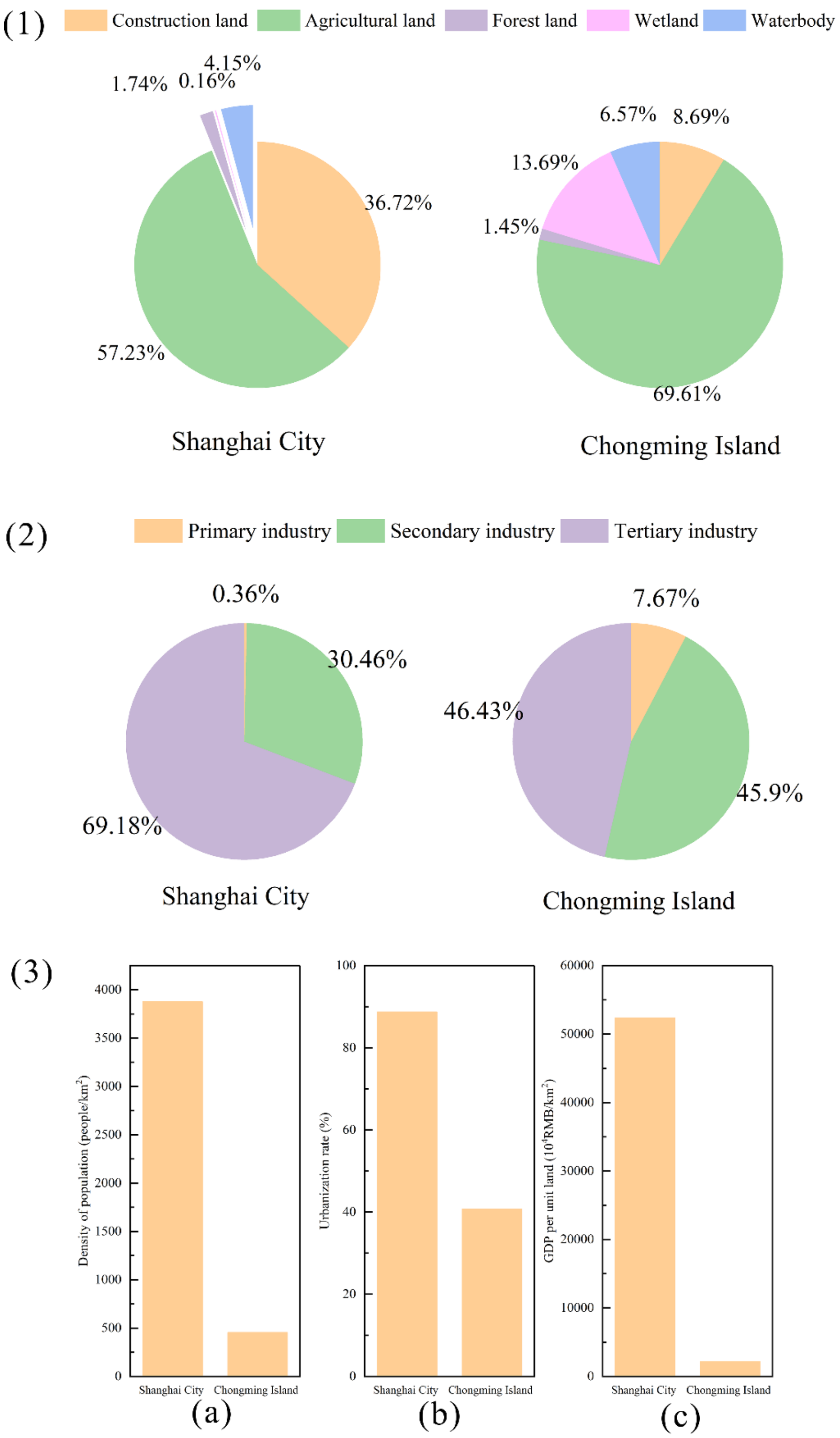

Figure S1. Comparison between Shanghai City and Chongming Island: (**1**) The proportion of land use; (**2**) The proportion of the first, second and third industries; (**3**) **a**. Density of population, **b.** urbanization rate, **c.** GDP per unit land.

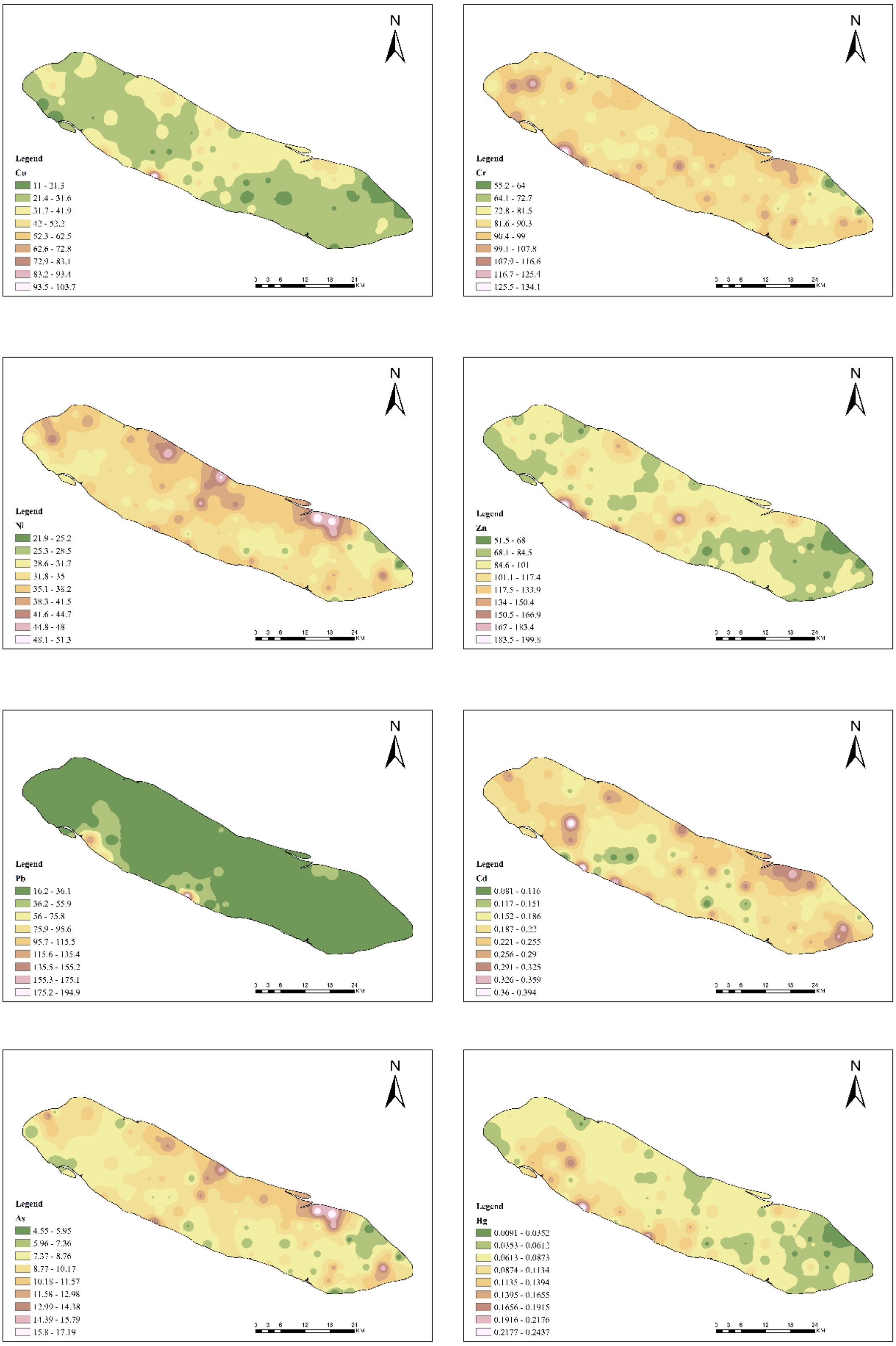

Figure S2. Distribution map of the average concentration of heavy metals (Cu, Cr, Ni, Zn, Pb, Cd, As and Hg) in Chongming Island.

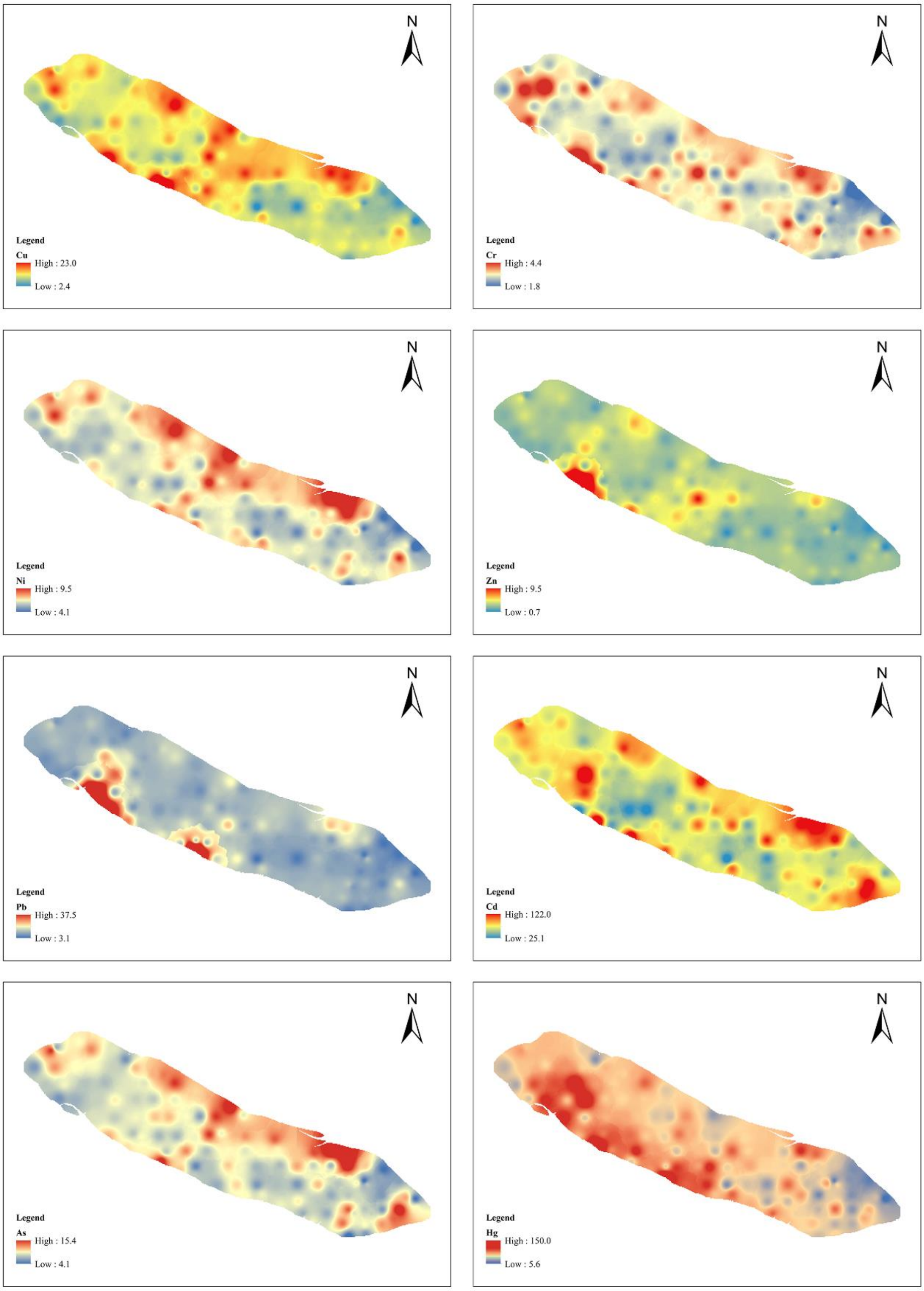

Figure S3. Distribution map of E_r^i values of heavy metals (Cu, Cr, Ni, Zn, Pb, Cd, As and Hg) in Chongming Island

References

1. USEPA.; Supplemental Guidance for Developing Soil Screening Levels for Superfund Sites. *Soild Waste Emerg. Responsewashingtondc* **2001**, *9355*, 9324–9354.
2. BMAQTS, Environmental site assessment guideline (DB11/T 656–2009). Beijing municipal administration of quanlity and technology supervision, Beijing, China, 2009.
3. CEPA, Environmental Quality Standard for Soils (GB15618–1995). In Administration, C.E.P.; European Policy Centre: Beograd, Srbija, 1995.
4. USEPA, Child-specific Exposure Factors Handbook. EPA-600-P-00e002B.National Center for Environmental Assessment; EPA: Washington, DC, USA, 2002.
5. Zeng, S.Y.; Ma, J.; Yang, Y.J.; Zhang, S.L.; Liu, G.J.; Chen, F.; Spatial assessment of farmland soil pollution and its potential human health risks in China. *Sci. Total Environ.* **2019**, *687*, 642–653.
6. USEPA, Exposure Factors Handbook. Office of Research and Development, National Center for Environmental Assessment, U.S. Environmental Protection Agency: Washington, DC, USA, 2011.
7. Cechinel, M.A.P.; Mayer, D.A.; Pozdniakova, T.A.; Mazur, L.P.; Boaventura, R.A.R.; de Souza, A.A.U.; de Souza, S.M.A.G.U.; Vilar, V.J.P.; Removal of metal ions from a petrochemical wastewater using brown macro-algae as natural cation-exchangers. *Chem. Eng. J.* **2016**, *286*, 1–15.
8. Men, C.; Liu, R.; Xu, F.; Wang, Q.; Guo, L.; Shen, Z.; Pollution characteristics, risk assessment, and source apportionment of heavy metals in road dust in Beijing, China. *Sci. Total Environ.* **2018**, *612*, 138–147.
9. Liang, J.; Feng, C.T.; Zeng, G.M.; Gao, X.; Zhong, M.Z.; Li, X.D.; Li, X.; He, X.Y.; Fang, Y.L.; Spatial distribution and source identification of heavy metals in surface soils in a typical coal mine city, Lianyuan, China. *Environ. Pollut.* **2017**, *225*, 681–690.
10. Lin, Y.; Ma, J.; Zhang, Z.D.; Zhu, Y.F.; Hou, H.; Zhao, L.; Sun, Z.J.; Xue, W.J.; Shi, H.D.; Linkage between human population and trace elements in soils of the Pearl River Delta: Implications for source identification and risk assessment. *Sci. Total Environ.* **2018**, *610*, 944–950.
11. Shazili, N.A.M.; Yunus, K.; Ahmad, A.S.; Abdullah, N.; Abd Rashid, M.K.; Heavy metal pollution status in the Malaysian aquatic environment. *Aquat. Ecosyst. Health Manage.* **2006,** 9, 137–145.
12. Xiao, R.; Guo, D.; Ali, A.; Mi, S.S.; Liu, T.; Ren, C.Y.; Li, R.H.; Zhang, Z.Q.; Accumulation, ecological-health risks assessment, and source apportionment of heavy metals in paddy soils: A case study in Hanzhong, Shaanxi, China. *Environ. Pollut.* **2019**, *248*, 349–357.
13. Yang, Y.; Christakos, G.; Guo, M.; Xiao, L.; Huang, W.; Space-time quantitative source apportionment of soil heavy metal concentration increments. *Environ. Pollut.* **2017**, *223*, 560–566.
14. Chen, T.; Chang, Q.; Liu, J.; Clevers, J.G.P.W.; Kooistra, L.; Identification of soil heavy metal sources and improvement in spatial mapping based on soil spectral information: A case study in northwest China. *Sci. Total Environ.* **2016**, *565*, 155–164.
15. Guan, Q.Y.; Wang, F.F.; Xu, C.Q.; Pan, N.H.; Lin, J.K.; Zhao, R.; Yang, Y.Y.; Luo, H.P.; Source apportionment of heavy metals in agricultural soil based on PMF: A case study in Hexi Corridor, northwest China. *Chemosphere* **2018**, *193*, 189–197.
16. Li, X.H.; Tang, Z.L.; Chu, F.Y.; Yang, L.Y.; Characteristics of distribution and chemical speciation of heavy metals in environmental mediums around Jinchang mining city, Northwest China. *Environ. Earth Sci.* **2011,** *64*, 1667–1674.
17. Pan, L.B.; Ma, J.; Wang, X.L.; Hou, H.; Heavy metals in soils from a typical county in Shanxi Province, China: Levels, sources and spatial distribution. *Chemosphere* **2016**, *148*, 248–254.
18. Qu, M.-K.; Li, W.-D.; Zhang, C.-R.; Wang, S.-Q.; Yang, Y.; He, L.-Y.; Source Apportionment of Heavy Metals in Soils Using Multivariate Statistics and Geostatistics. *Pedosphere* **2013,** *23*, 437–444.
19. Aminiyan, M.M.; Baalousha, M.; Mousavi, R.; Aminiyan, F.M.; Hosseini, H.; Heydariyan, A.; The ecological risk, source identification, and pollution assessment of heavy metals in road dust: A case study in Rafsanjan, SE Iran. *Environ. Sci. Pollut. Res.* **2018,** *25*, 13382–13395.
20. Wannaz, E.D.; Carreras, H.A.; Rodriguez, J.H.; Pignata, M.L.; Use of biomonitors for the identification of heavy metals emission sources. *Ecol. Indic.* **2012**, *20*, 163–169.
21. Belon, E.; Boisson, M.; Deportes, I.Z.; Eglin, T.K.; Feix, I.; Bispo, A.O.; Galsomies, L.; Leblond, S.; Guellier, C.R.; An inventory of trace elements inputs to French agricultural soils. *Sci. Total Environ.* **2012**, *439*, 87–95.
22. Nicholson, F.A.; Smith, S.R.; Alloway, B.J.; Carlton-Smith, C.; Chambers, B.J.; An inventory of heavy metals inputs to agricultural soils in England and Wales. *Sci. Total Environ.* **2003,** *311*, 205–219.

23. Al-Wabel, M.I.; Sallam, A.S.; Usman, A.R.A.; Ahmad, M.; El-Naggar, A.H.; El-Saeid, M.H.; Al-Faraj, A.; El-Enazi, K.; Al-Romian, F.A.; Trace metal levels, sources, and ecological risk assessment in a densely agricultural area from Saudi Arabia. *Environ. Monit. Assess.* **2017,** *189*, 21.
24. Calvo, A.I.; Alves, C.; Castro, A.; Pont, V.; Vicente, A.M.; Fraile, R.; Research on aerosol sources and chemical composition: Past, current and emerging issues. *Atmos. Res.* **2013**, *120*, 1–28.
25. Huang, Y.; Li, T.Q.; Wu, C.X.; He, Z.L.; Japenga, J.; Deng, M.; Yang, X.; An integrated approach to assess heavy metal source apportionment in pen-urban agricultural soils. *J. Hazard. Mater.* **2015**, *299*, 540–549.
26. Saeedi, M.; Li, L.Y.; Salmanzadeh, M.; Heavy metals and polycyclic aromatic hydrocarbons: Pollution and ecological risk assessment in street dust of Tehran. *J. Hazard. Mater.* **2012**, *227*, 9–17.
27. Sun, L.; Guo, D.K.; Liu, K.; Meng, H.; Zheng, Y.J.; Yuan, F.Q.; Zhu, G.H.; Levels, sources, and spatial distribution of heavy metals in soils from a typical coal industrial city of Tangshan, China. *Catena* **2019**, *175*, 101–109.
28. Yadav, I.C.; Devi, N.L.; Singh, V.K.; Li, J.; Zhang, G.; Spatial distribution, source analysis, and health risk assessment of heavy metals contamination in house dust and surface soil from four major cities of Nepal. *Chemosphere* **2019**, *218*, 1100–1113.
29. Luo, X.-S.; Xue, Y.; Wang, Y.-L.; Cang, L.; Xu, B.; Ding, J.; Source identification and apportionment of heavy metals in urban soil profiles. *Chemosphere* **2015**, *127*, 152–157.
30. Wang, C.; Yang, Z.; Zhong, C.; Ji, J.; Temporal-spatial variation and source apportionment of soil heavy metals in the representative river-alluviation depositional system. *Environ. Pollut.* **2016**, *216*, 18–26.
31. Tang, Z.; Chai, M.; Cheng, J.; Jin, J.; Yang, Y.; Nie, Z.; Huang, Q.; Li, Y.; Contamination and health risks of heavy metals in street dust from a coal mining city in eastern China. *Ecotoxicol. Environ. Saf.* **2017**, *138*, 83–91.
32. Yildirim, G.; Tokalioglu, S.; Heavy metal speciation in various grain sizes of industrially contaminated street dust using multivariate statistical analysis. *Ecotoxicol. Environ. Saf.* **2016**, *124*, 369–376.
33. Duan, J.; Tan, J.; Atmospheric heavy metals and Arsenic in China: Situation, sources and control policies. *Atmos. Environ.* **2013**, *74*, 93–101.
34. Liang, J.; Feng, C.; Zeng, G.; Zhong, M.; Gao, X.; Li, X.; He, X.; Li, X.; Fang, Y.; Mo, D.; Atmospheric deposition of mercury and cadmium impacts on topsoil in a typical coal mine city, Lianyuan, China. *Chemosphere* **2017**, *189*, 198–205.
35. Cloquet, C.; Carignan, J.; Libourel, G.; Sterckeman, T.; Perdrix, E.; Tracing source pollution in soils using cadmium and lead isotopes. *Environ. Sci. Technol.* **2006,** *40*, 2525–2530.
36. Lu, A.X.; Wang, J.H.; Qin, X.Y.; Wang, K.Y.; Han, P.; Zhang, S.Z.; Multivariate and geostatistical analyses of the spatial distribution and origin of heavy metals in the agricultural soils in Shunyi, Beijing, China. *Sci. Total Environ.* **2012**, *425*, 66–74.
37. Chi Thanh, V.; Lin, C.; Shern, C.-C.; Yeh, G.; Le, V.G.; Huu Tuan, T.; Contamination, ecological risk and source apportionment of heavy metals in sediments and water of a contaminated river in Taiwan. *Ecol. Indic.* **2017**, *82*, 32–42.
38. Lv, J.S.; Wang, Y.M.; Multi-scale analysis of heavy metals sources in soils of Jiangsu Coast, Eastern China. *Chemosphere* **2018**, *212*, 964–973.
39. Xie, R.; Seip, H.M.; Wibetoe, G.; Nori, S.; McLeod, C.W.; Heavy coal combustion as the dominant source of particulate pollution in Taiyuan, China, corroborated by high concentrations of arsenic and selenium in PM10. *Sci. Total Environ.* **2006,** *370*, 409–415.
40. Fernandez, S.; Cotos-Yanez, T.; Roca-Pardinas, J.; Ordonez, C.; Geographically Weighted Principal Components Analysis to assess diffuse pollution sources of soil heavy metal: Application to rough mountain areas in Northwest Spain. *Geoderma* **2018**, *311*, 120–129.
41. Qu, M.; Wang, Y.; Huang, B.; Zhao, Y.; Source apportionment of soil heavy metals using robust absolute principal component scores-robust geographically weighted regression (RAPCS-RGWR) receptor model. *Sci. Total Environ.* **2018**, *626*, 203–210.
42. Zhang, P.; Qin, C.; Hong, X.; Kang, G.; Qin, M.; Yang, D.; Pang, B.; Li, Y.; He, J.; Dick, R.P.; Risk assessment and source analysis of soil heavy metal pollution from lower reaches of Yellow River irrigation in China. *Sci. Total Environ.* **2018**, *633*, 1136–1147.
43. Atafar, Z.; Mesdaghinia, A.; Nouri, J.; Homaee, M.; Yunesian, M.; Ahmadimoghaddam, M.; Mahvi, A.H.; Effect of fertilizer application on soil heavy metal concentration. *Environ. Monit. Assess.* **2010,** *160*, 83–89.

44. Cai, L.M.; Wang, Q.S.; Wen, H.H.; Luo, J.; Wang, S.; Heavy metals in agricultural soils from a typical township in Guangdong Province, China: Occurrences and spatial distribution. *Ecotoxicol. Environ. Saf.* **2019**, *168*, 184–191.
45. Li, S.Y.; Jia, Z.M.; Heavy metals in soils from a representative rapidly developing megacity (SW China): Levels, source identification and apportionment. *Catena* **2018**, *163*, 414–423.
46. Liu, X.; Zhang, L.; Concentration, risk assessment, and source identification of heavy metals in surface sediments in Yinghai: A shellfish cultivation zone in Jiaozhou Bay, China. *Mar. Pollut. Bull.* **2017,** *121,* 216–221.
47. Streets, D.G.; Hao, J.M.; Wu, Y.; Jiang, J.K.; Chan, M.; Tian, H.Z.; Feng, X.B.; Anthropogenic mercury emissions in China. *Atmos. Environ.* **2005,** *39*, 7789–7806.

© 2020 by the authors. Licensee MDPI, Basel, Switzerland. This article is an open access article distributed under the terms and conditions of the Creative Commons Attribution (CC BY) license (http://creativecommons.org/licenses/by/4.0/).
